# Supplementary material for: Crystal-chemical origins of the ultrahigh conductivity of metallic delafossites
Source: Nat Commun. 2024 Feb 15;15:1399. doi: 10.1038/s41467-024-45239-6 (PMC10869826; doi:10.1038/s41467-024-45239-6)
Supplement: Supplementary file 1 — Supplementary Information [file 41467_2024_45239_MOESM1_ESM.docx]

Supplementary Information

**Crystal-Chemical Origins of the Ultrahigh Conductivity
of Metallic Delafossites**

Yi Zhang (張易), Fred Tutt, Guy N. Evans, Prachi Sharma, Greg Haugstad, Ben Kaiser,

Justin Ramberger, Samuel Bayliff, Yu Tao, Mike Manno, Javier Garcia-Barriocanal, Vipul Chaturvedi, Rafael M. Fernandes, Turan Birol, William E. Seyfried Jr., and Chris Leighton*

*Corresponding author. Email: leighton@umn.edu

**Supplementary Note 1. Chemical Vapor Transport (CVT) Crystal Growth**

***CVT Setup and Optimization*.** As noted in the main text, while CVT has not been reported for PdCoO_2_, there are several reasons to consider this growth approach, including the fact that PdCoO_2_ decomposes^1–3^ and that PdCl_2_ (which decomposes at ~600 °C in low Cl pressure^4,5^) is a potentially convenient transport agent. CVT crystal growth of Co_3_O_4_^6^, Pd^6^, and PdO^6^ have also been reported. A CVT process was thus designed based on the known properties of PdCoO_2_, PdCl_2_, and CoCl_2_.

CVT growth of Co_3_O_4_, Pd, and PdO using PdCl_2_ as a transport agent have been reported at growth temperatures in the 400-1000 °C range^6^, based on reactions such as ${Co}_{3}O_{4}\left( s \right)+3{Cl}_{2}\left( g \right)\rightleftharpoons3{CoCl}_{2}\left( g \right)+2O_{2}\left( g \right)$, $Pd\left( s \right)+{Cl}_{2}\left( g \right)\rightleftharpoons{PdCl}_{2}\left( g \right)$, and $2PdO\left( s \right)+{2Cl}_{2}\left( g \right)\rightleftharpoons{2PdCl}_{2}\left( g \right)+O_{2}(g)$. The decomposition of PdCoO_2_ in air commences at ~800 °C^3^, in the range of established CVT temperatures for Co_3_O_4_, Pd, and PdO^6^. We therefore propose the analogous transport reaction, ${PdCoO}_{2}\left( s \right)+2{Cl}_{2}\left( g \right)\rightleftharpoons{CoCl}_{2}\left( g \right)+{PdCl}_{2}\left( g \right)+O_{2}(g)$, where, in the forward reaction, solid PdCoO_2_ is volatilized by Cl_2_ vapor generated from partial thermal decomposition of PdCl_2_, then transformed into a mixture of gaseous CoCl_2_, PdCl_2_, and O_2_ at the hot end of the growth ampoule. CoCl_2_ and PdCl_2_ vapors then transport across the temperature gradient, forming PdCoO_2_ in the ampoule’s cold end through the reverse reaction.

The choice of transport agent was also considered, *i.e*., whether to use PdCl_2_, PdBr_2_, or PdI_2_, for example. For PdCoO_2_ growth, the co-stability of halides of Co and Pd is essential. PdBr_2_ and PdI_2_ have very low decomposition points (250 and 360 °C, respectively^7^), much lower than the CoBr_2_ and CoI_2_ melting points (678 and 520 °C, respectively)^7^. The bromides or iodides of Pd and Co having comparable vapor pressures at reasonable temperatures is thus unlikely. PdCl_2_, however, decomposes at 600-740 °C (the range is likely due to variable Cl_2_ pressures)^4,5^, with a melting point at 680 °C^7^, while CoCl_2_ has a similar melting point of 737 °C^7^; this suggests a reasonable likelihood of co-stability of their vapors at ~700 °C. PdCl_2_ was therefore chosen as the transport agent, taking advantage of its partial thermal decomposition to generate Cl_2_ at ~600 °C, without the use of hazardous Cl_2_ gas, and avoiding potential impurities from Cl_2_-generating transport agents such as HgCl_2_^6^.

As shown in Supplementary Fig. 1a, for CVT growth, vacuum-sealed quartz ampoules (see Methods) were held horizontally in a multi-zone furnace, with the precursor powders (~1 g of metathesis/flux-grown PdCoO_2_ and ~0.12g of PdCl_2_) loaded at the right end of the ampoule (in Zone 2), and the empty end of the ampoule in Zone 1. For the first 3 days, Zone 1 was the hot zone and Zone 2 the cold zone, while for the remaining 13 days the temperatures gradient was reversed; the rationale for this is provided below. A typical ampoule after the end of a CVT growth is shown in Supplementary Fig. 1b. At the end of a successful growth, essentially all of the PdCoO_2_ is transported to the growth (cold) zone, forming PdCoO_2_ single crystals, PdCoO_2_ multicrystals, and excess chlorides. The other ampoule end is essentially empty, indicating high yield.

A 50 °C temperature difference was chosen for most growths in this work. Smaller differences, such as 30 °C, led to slow crystal growth rates and incomplete transport in 13 days, while larger differences, such as 60 °C, led to smaller crystal sizes, likely indicating too high a transport rate. Most growths were done with 710/760 °C cold/hot zone temperatures (such as the ampoule in Supplementary Fig. 1b), which were found to yield the largest crystals, with reflective surfaces, and near-complete transport. Higher temperature conditions were experimented with, using 750/800 °C and 800/850 °C (the ampoules in Supplementary Figs. 1c,d, respectively), for example. At these higher temperatures, the yield of PdCoO_2_ crystals decreased, with no improvement in crystal size. A magenta coating also developed on the inner ampoule walls, as in metathesis/flux growth at ≥750 °C, which was found by X-ray diffraction to be mainly Co_2_SiO_4_. This likely results from high-temperature side reactions between cobalt compounds and quartz. Eventually, in the 800/850°C growth, no PdCoO_2_ crystals were grown, and PdO, along with <10 wt.% untransported PdCoO_2_ precursor was found to remain in the hot zone. At these temperatures, PdCoO_2_ likely decomposes into binaries at 850 °C, the resulting cobalt compounds reacting with the quartz to form the Co_2_SiO_4_ tube coating in Supplementary Fig. 1d.

***CVT Mechanisms.*** Based on the above, and on additional observations below, various conclusions can be reached regarding the mechanisms of CVT growth. The growth starts with the initial conditions in Supplementary Fig. 2a, with the mix of precursor powders located in Zone 2 and the nominally empty ampoule end in Zone 1. Note here that the “empty” end of the ampoule is inevitably unintentionally dusted with precursor powder particles during loading, as shown in Supplementary Fig. 2a. As it is common for metathesis/flux-grown PdCoO_2_ (one of the precursors here) to have small amounts of impurity phases, including Pd (see Supplementary Note 2 below), there are thus microscopic quantities of Pd present, as also shown in Supplementary Fig. 2a. In the first stage of the growth, illustrated in Supplementary Fig. 2b, the ampoule end filled with precursors is the cold zone (Zone 2) and the empty end is the hot zone (Zone 1), for an inverted-temperature-gradient period of 3 days. The purpose of this first stage is to transport any PdCoO_2_ powder particles in the “empty” end of the ampoule to the other end, hence “cleaning” the growth zone, *i.e*., ridding it of stray nuclei. During this stage however, which was found to be essential, Pd (whether from phase impurities in the metathesis/flux-grown PdCoO_2_ or partial thermal decomposition of PdCl_2_) will be transported to the empty ampoule end, as Pd is known to transport *via* Cl_2_ from cool to hot ^6^; this is due to the exothermic $Pd\left( s \right)+{Cl}_{2}\left( g \right)\to{PdCl}_{2}\left( g \right)$ reaction^6^. The significance of this point is returned to below. The initial 3 days of inverted temperature gradient are followed by the main (13-day) growth stage shown in Supplementary Fig. 2c. At the start of this period, as explained above, the growth zone (Zone 1) can be expected to contain Pd crystallites (whether there originally, or transported from Zone 2). These Pd crystallites may then act as nucleation centers for PdCoO_2_ crystal growth. Notably, the interatomic distance within the (111) planes of Pd is 0.275 nm^8^, similar (within -2.8%) to the PdCoO_2_ lattice parameter of *a* = 0.283 nm^1,8,9^. Pd particles can thus likely seed the PdCoO_2_ CVT crystal growth in this process, which we propose as an explanation for the multicrystal formation that we found common. In essence, we propose that PdCoO_2_ crystals can grow in multiple directions from different (111)-family facets of Pd crystallites, generating multicrystals. As noted in the main text, these multicrystals can be easily separated into single crystals, and some single crystals also form, presumably due to non-Pd-seeded growth. As a final comment on this mechanism, note that PdO may also play a similar role in nucleation.

**
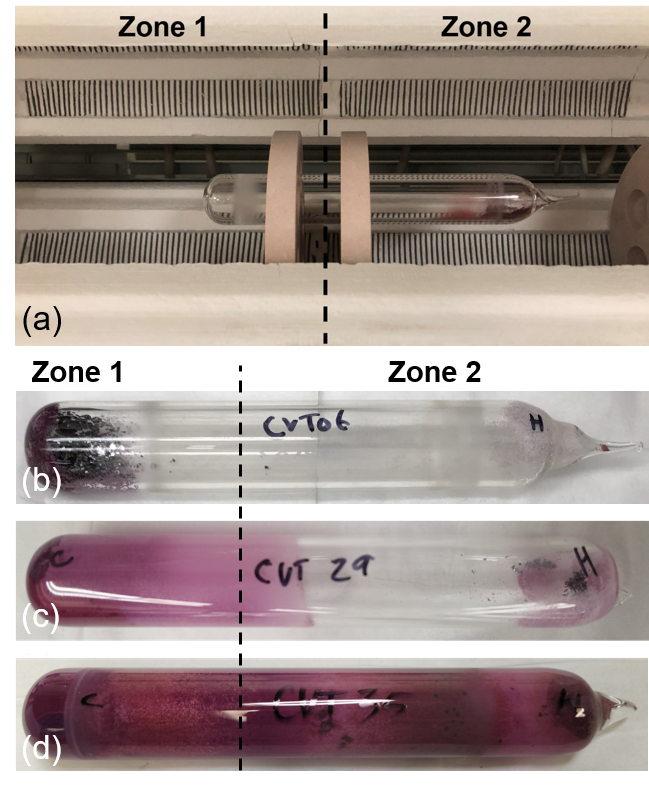
**

**Supplementary Fig. 1. CVT growth setup and example ampoules.** (a) Two-zone furnace setup prior to CVT growth. The ~6-inch-long quartz ampoule is held between two zones labeled Zone 1 and Zone 2, with the boundary marked by the black dashed line. The precursor powders are loaded into the right side of the ampoule, in Zone 2. (b) Example quartz ampoule after a 710/760 °C CVT growth. “Zone 1”, “Zone 2”, and the dashed line mark the in-furnace position of the ampoule. CVT crystals are grown in the left side of the ampoule, in Zone 1, at essentially complete yield. (c) Example quartz ampoule after a 750/800 °C CVT growth. (d) Example quartz ampoule after a 800/850 °C growth. In (c,d), note the magenta coating inside the quartz tubes, identified as Co_2_SiO_4_.


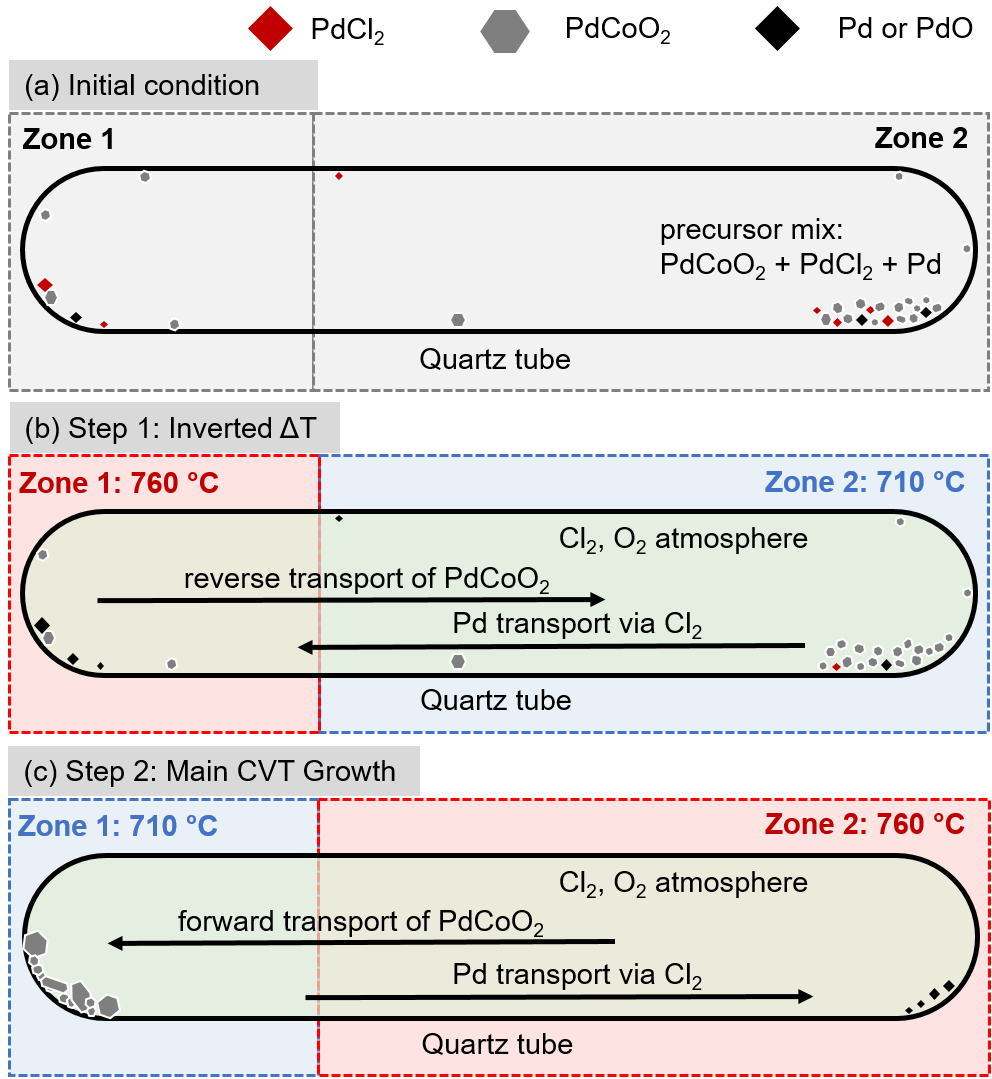


**Supplementary Fig. 2. Schematics illustrating various stages of PdCoO_2_ CVT growth.** Growth ampoules after (a) initial loading, (b) the first, inverted-temperature-gradient stage (3 days), and (c) the second, main growth stage (13 days). The legend at the top identifies PdCl_2_, PdCoO_2_, and Pd/PdO. In (a), the majority of the precursors (PdCoO_2_ and PdCl_2_ powder) are in Zone 2 but stray powder inevitably populates Zone 1. Pd is present only as an impurity in the metathesis/flux-grown PdCoO_2_. In (b), during the inverted-temperature-gradient stage, Zone 1 is at 760 °C and Zone 2 is at 710 °C. The PdCl_2_ has partially decomposed into Pd and Cl_2_, and Cl_2_ in turn reacts with PdCoO_2_ to form PdCl_2_, CoCl_2_, and O_2_. PdCoO_2_ is transported from Zone 1 to 2 (removing stray nuclei) but microscopic amounts of Pd (and possibly PdO) are transported from Zone 2 to 1. In (c), during the main growth stage, Zone 1 is at 710 °C and Zone 2 is at 760 °C. PdCoO_2_ is transported from Zone 2 to Zone 1 and Pd is transported from Zone 1 to Zone 2.

**Supplementary Note 2. Specific Growth Parameters of Characterized Crystals**

Supplementary Tables 1 and 2 summarize the metathesis/flux and CVT growth conditions used to grow the specific PdCoO_2_ crystals characterized in this paper. Powder X-ray diffraction (PXRD) was performed on almost all metathesis/flux and CVT crystals, and the other characterization/ measurement techniques applied are listed in each case.

In addition to the characterization in the main text, Supplementary Fig. 3 shows additional PXRD from four further metathesis/flux crystal batches and four further CVT batches. The phase purity is seen to significantly improve *via* CVT. As shown in Supplementary Fig. 3a, Co_3_O_4_ and Pd minor phase impurities were commonly found in our metathesis/flux crystals. In CVT crystals (Supplementary Fig. 3b), only a minor Pd phase impurity was ever detected.

**Supplementary Table 1. Growth parameters of metathesis/flux-grown crystals.** Crystal growth parameters of metathesis/flux growth batches that produced samples characterized in this paper. Each batch corresponds to one ampoule. Listed are the batch numbers, growth temperature (*T*_growth_), dwell time at *T*_growth_, cooling rate, and characterization methods. Characterizations that appear in the main text are bolded with the corresponding figure/table in parentheses. PXRD is powder X-ray diffraction, SEM is scanning electron microscopy, and ICP-MS is inductively coupled plasma mass spectrometry.

| **Batch number** | ***T*_growth_**  **(°C)** | **Dwell time at *T*_growth_ (h)** | **Cooling rate**  **(°C/h)** | **Characterized by:** |
| --- | --- | --- | --- | --- |
| M/F06 | 700 | 40 | 60 | PXRD, **SEM** (Fig. 1b),  **magnetometry** (Table 1) |
| M/F62 | 750 | 150 | 40 | PXRD, **optical** **imaging** (Fig. 1b, inset) |
| M/F29 | 750 | 60 | 40 | **PXRD** (Fig. 1d) |
| M/F30 | 750 | 60 | 40 | **PXRD** (Fig. 1d) |
| M/F55 | 750 | 100 | 40 | PXRD, **ICP-MS** (Tables 2-4) |
| M/F56 | 750 | 100 | 40 | PXRD, **ICP-MS** (Tables 2-4) |
| M/F59 | 750 | 150 | 40 | PXRD, **ICP-MS** (Tables 2-4) |

**Supplementary Table 2. Growth parameters of CVT-grown crystals.** Crystal growth parameters of CVT batches that produced samples characterized in this paper. Each batch corresponds to one ampoule. Listed are the batch numbers, cold and hot zone temperatures (*T*_cold_ and *T*_hot_), and characterization methods. Characterizations that appear in the main text are bolded with the corresponding figure/table in parentheses. PXRD is powder X-ray diffraction, ICP-MS is inductively coupled plasma mass spectrometry, PIXE is particle-induced X-ray emission, EDS is energy-dispersive X-ray spectroscopy, HRXRD is high-resolution X-ray diffraction, and RC is X-ray rocking curve analysis.

| **Batch number** | ***T*_cold_**  **(°C)** | ***T*_hot_**  **(°C)** | **Characterized by:** |
| --- | --- | --- | --- |
| CVT34 | 710 | 760 | PXRD, **optical imaging** (Fig. 1c inset), **ICP-MS** (Fig. 4c, Tables 2-4) |
| CVT14 | 710 | 760 | PXRD, **optical imaging** (Fig. 1c), **PIXE** (Fig. 2e) |
| CVT12 | 710 | 760 | **PXRD** (Fig. 1e), optical imaging, **EDS** (Fig. 2d), transport |
| CVT29 | 750 | 800 | PXRD, optical imaging, **Laue** (Fig. 2a), **HRXRD** (Fig. 2c),  **RC** (Fig. 2c inset) |
| CVT24 | 710 | 760 | PXRD, optical imaging, **PIXE** (Fig. 2e), **ICP-MS** (Fig. 4c, Tables 2-4), magnetometry |
| CVT25 | 710 | 760 | PXRD, optical imaging, **PIXE** (Fig. 2e), **ICP-MS** (Fig. 4c, Tables 2-4), **transport** (Figs. 3a,b), VSM |
| CVT01 | 680 | 740 | PXRD, calorimetry, **magnetometry** (Fig. 3c, d, Table 1), EDS |
| CVT33 | 710 | 760 | PXRD, **ICP-MS** (Fig. 4c, Tables 2-4), PIXE |

**
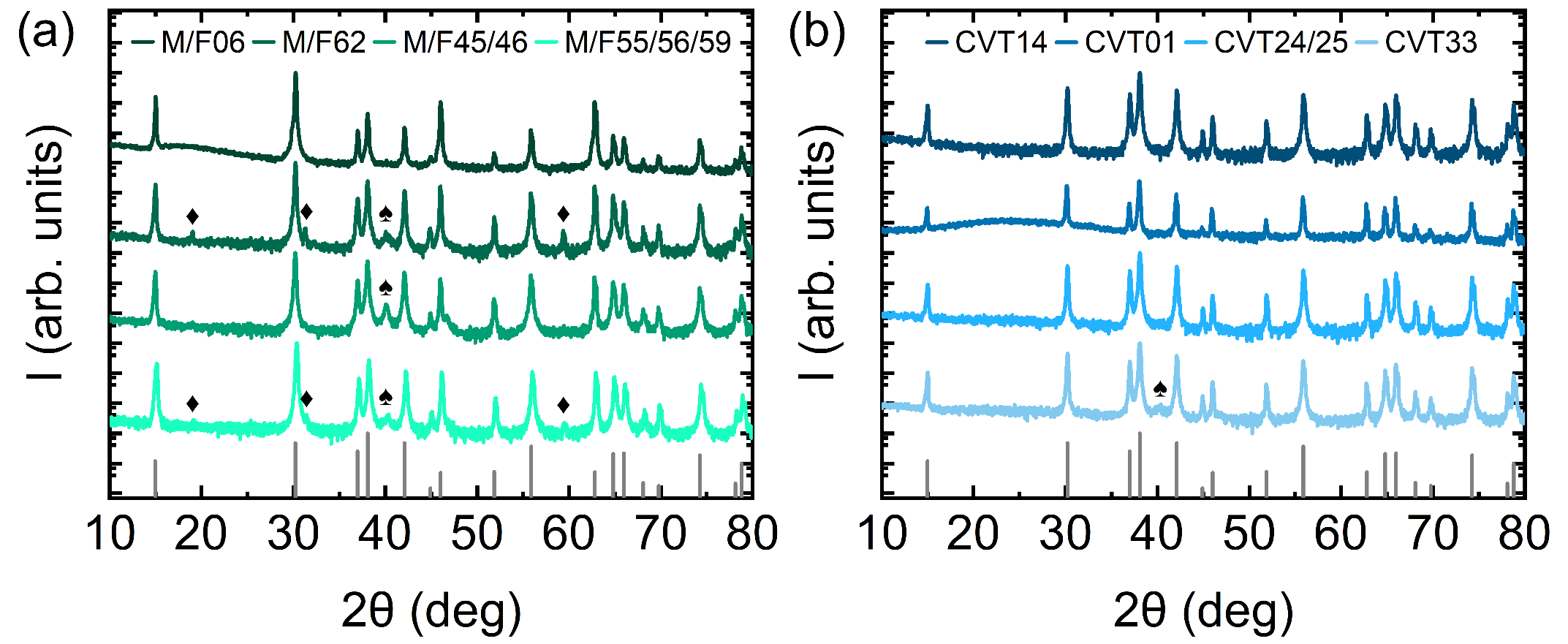
**

**Supplementary Fig. 3. Additional PXRD data from metathesis/flux and CVT crystals.** PXRD scans (intensity *I* *vs*. angle 2θ) from (a) four metathesis/flux crystal batches (green) and (b) four CVT crystal batches (blue). Peaks due to Co_3_O_4_ and Pd phase impurities are marked with diamond and spade symbols, respectively. PdCoO_2_ reference patterns are shown at the bottom in gray^1,10^.

**Supplementary Note 3. Transport Measurement Details**

***Offset Correction and Signal-to-noise Ratio***. As noted in the main text, the very low residual value of the *a-b* plane resistivity (ρ_ab_) in PdCoO_2_ single crystals renders low-temperature (*T*) transport measurements challenging. Specifically, signal-to-noise ratio becomes an issue, as do instrumental offsets. These issues were alleviated in this work through the study of relatively long bar-shaped crystals (Fig. 3a, inset), but must still be carefully addressed. The signal-to-noise ratio was maximized through the use of an AC resistance bridge (a Lakeshore 370 operating at 13.7 Hz) in tandem with a preamplifier/channel scanner (a Lakeshore 3708). Currents of 10 mA were employed and self-heating was rigorously ruled out by estimations of the (very low) deposited power, and careful low-*T* checks of resistance *vs*. current and resistance *vs*. *T* at various excitation currents. The resulting noise performance is illustrated below and in the data of Figs. 3a, b.

With respect to offsets, which are critical given the ~20 μΩ residual resistances, these were first carefully characterized *via* parallel measurements of zero-resistance superconducting V thin films. The latter were Si/Si-N/V(100 nm) films deposited in ultrahigh vacuum in a molecular beam epitaxy system. As shown in the inset to Supplementary Fig. 4, the same top-contact, in-line, four-wire geometry as was used for PdCoO_2_ crystals (Fig. 3a, inset) was employed for the V films. The four contacts are labeled A, B, C, D, and a standard notation for four-wire resistance (*R*) measurements is followed by using the first two letters to refer to the *I*+ and *I*- terminals, respectively, and the last two letters to refer to the *V*+ and *V*- terminals, respectively, *e.g*., *R*_ADBC_. As shown in Supplementary Fig. 4, the nominally zero-magnetic-field *R_ADBC_*(*T*) of such films reveals a superconducting transition with an onset temperature of ~5.2 K and an endpoint of ~5.1 K, in agreement with expectations^11^. Such samples were thus measured at 3.9 K, safely in the zero-resistance regime, to characterize the voltage and resistance offsets in our measurement set-up.

Offset measurements were made both with and without the preamplifying channel scanner. Supplementary Table 3 shows typical results for two separate sets of contacts on V films at 3.9 K. The first three data columns are without the preamplifier, and the last three data columns are with the preamplifier. Considering the data without the preamplifier first, measurements of *R*_ADBC_ and *R*_DACB_ were found to be near identical, at -0.4 to -0.3 μΩ for Contact Set 1 and 0.3 to 0.7 μΩ for Contact Set 2. Such findings are representative. Over many measurements, using different wire and contact sets, different bridge ranges and currents (10 mA was used in Supplementary Table 3), *etc*., the resistances offsets from zero were found to be approximately ±1 μΩ, with noise of approximately ±0.5 μΩ. Most importantly, the approximately ±1 μΩ offset indicates that measurements of ~20 μΩ resistance can be made with reasonable accuracy. Adding the preamplifier to the set-up (right side of Supplementary Table 3) was found to result in differences. The noise dropped to approximately ±0.1 μΩ (by roughly a factor of 5), but the offsets from zero resistance were found to grow to approximately ±5 μΩ (also by roughly a factor of 5). Interestingly, however, it was found empirically that the sign of the offset reproducibly inverted on going from *R*_ADBC_ to *R*_DACB_, *i.e*., with a 180° phase shift of the excitation (Supplementary Table 3). Averaging between *R*_ADBC_ and *R*_DACB_ thus yielded offsets of only ±1-2 μΩ, over many measurements, using different wire and contact sets, different bridge ranges and currents, *etc.* Thus, regardless of whether the preamplifier was included, offsets from zero resistance could be kept to a workable ±1-2 μΩ.

Based on the above characterization of offsets in the measurement setup, PdCoO_2_ single-crystal measurements were made two ways. First, for the residual resistance measurement at ~4 K on the CVT-grown PdCoO_2_ crystal in the inset to Fig. 3a, the preamplifier was removed, resulting in *R*_ADBC_ = 19.4 μΩ as the average value, with a noise level of approximately ±0.5 μΩ. Accepting the ±1 μΩ offset from the above analysis, using the measured sample dimensions (0.0225 ± 0.0006 mm thick, 1.045 ± 0.005 mm long, and 0.20 ± 0.02 mm wide), and propagating errors then leads to a residual ρ_ab_ of 8 ± 1 nΩ cm. Combining this with the measured 300-K *R*_ADBC_ of 8465 ± 5 μΩ (where the offset is negligible) yields the RRR of 436 ± 25 quoted in the main text. Second, for low-noise ρ_ab_(*T*) measurements (see Figs. 3a, b), the preamplifier was inserted and both *R*_ADBC_(*T*) and *R*_DACB_(*T*) were measured, as shown in Supplementary Fig. 5. Based on the above analysis, these values were then averaged to the solid line in Supplementary Fig. 5, thereby accounting for the offsets to within ±1-2 μΩ.

***Finite-element Simulations of Current Flow and Voltage Drop.*** As noted in the main text, the large *c*-axis/*a-b*-plane resistivity ratio (ρ_c_/ρ_ab_) of PdCoO_2_ single crystals^9,12–15^ provides a second measurement challenge. Specifically, the top-contact geometry used here, while convenient, must lead to some level of additional systematic error due to some inevitable current flow along *c*-axis directions. Finite element current flow and voltage drop simulations were thus performed using COMSOL Multiphysics. For these simulations, samples with dimensions close to the crystal in Fig. 3a were set up, as shown in Supplementary Fig. 6. Two current injection geometries were studied. Supplementary Fig. 6a shows the top-contact geometry employed in our experiments, while Supplementary Fig. 6b shows a side-contact geometry for current injection. The former closely replicates the experimental geometry, while the latter illustrates the effect of more ideally constraining the current in the *a-b*-plane, thus minimizing the influence of the large ρ_c_/ρ_ab_. The boundary conditions applied in both geometries consisted of a 10 mA current flow from contact A, with a grounded contact D; an automatically generated mesh was used in the simulations.

For the purposes of initial illustration, Supplementary Figs. 6a,b, are simulation results based on the reported ρ_ab_^16^ and ρ_c_^13^ of metathesis/flux-grown single crystals (with RRR = 376), the ρ_ab_ values being from ion-beam-patterned crystals^16^. Although it impacts the results negligibly, the contacts were simulated using typical resistivities for Cu. All of the 300-K and low-*T* resistivity values are listed in Supplementary Table 4. As shown by Supplementary Fig. 6b, using 300-K values, the color maps of voltage drop in the side-current-contact geometry are as expected. No potential differences are apparent between the top and bottom of the crystal, and the voltage is dropped gradually from A to D. As shown by Supplementary Fig. 6a, however, this is not so when the all-top-contact geometry is simulated. Due to the large ρ_c_/ρ_ab_, distinct (~25%) potential differences arise from the top to the bottom of the crystal near the contacts, despite the thickness of only 22 μm. For quantification, the simulation results were then used to determine the 4-terminal resistance using *R* = (*V*_C_ - *V*_B_)/*I*. The results are summarized in Supplementary Table 4 (right columns) for the side-contact and top-contact geometries. The side-contact results are as expected, reproducing the input RRR from resistivity to good accuracy. The top-contact simulated *R* values are 50-90% larger than those for side current contacts, however, due to the effect of the large ρ_c_/ρ_ab_. This effect worsens with decreasing *T* as ρ_c_/ρ_ab_ grows on cooling (also shown in Supplementary Table 4). The top-contact RRR of 278 is thus ~20% smaller than the side-contact (true) value. As stated in the main text, measured RRR values in this work (~440 for CVT crystals) are thus lower bounds due to overestimation of the residual ρ_ab_.

Further quantification of the extent of underestimation of the RRR was achieved by running additional top-contact simulations that reproduce the observed RRR of ~440. This was done by finding the required true value of residual ρ_ab_ to reproduce the experimental results, while keeping the 300-K value of ρ_ab_ (which is dominated by phonon, not impurity, scattering) constant. (In the absence of additional information, ρ_c_ was also maintained at the same values as in prior simulations). As shown in Supplementary Table 5, the true residual ρ_ab_ required to reproduce a top-current-contact RRR of 436 is 4.4 nΩcm, close to half the measured value. The measured RRR of 436, by our best estimates, thus corresponds to a true value of 670 (Supplementary Table 5). As noted in the main text, these estimates are further supported by unitary scattering limit calculations, which indicate that our measured Pd-plane impurity density is consistent with this residual ρ_ab_ to within 15-30%. Note that the above simulations assume ρ_c_ is unchanged from metathesis/flux crystals to CVT crystals. If we were to assume some reduction in ρ_c_, which is likely based on the higher purity, then the ρ_ab_ needed to reproduce the measured top-contact RRR would increase. The RRR estimate of 670 should thus be viewed as an upper bound for the true value, yielding 440 < RRR < 670.


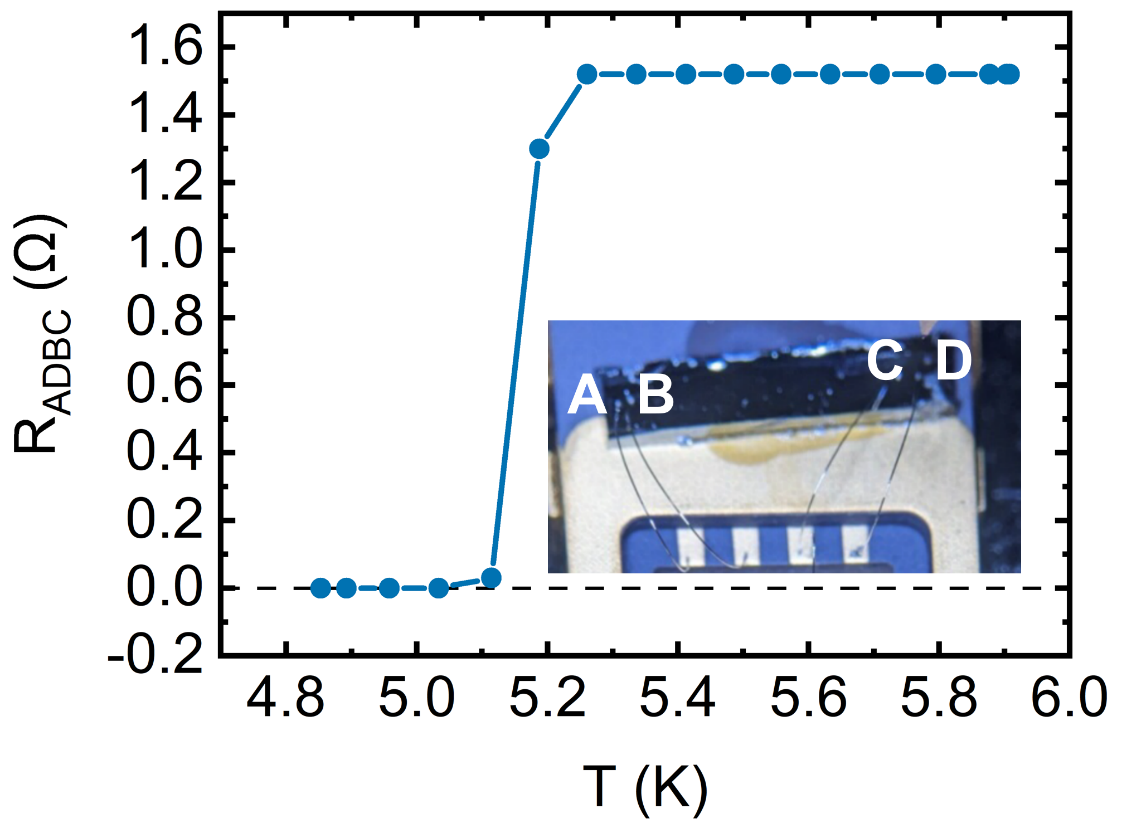


**Supplementary Fig. 4. Superconducting transition of V thin films used for offset characterization.** *R*_ADBC_(*T*) (see above text for definition) of a Si/Si-N/V(100 nm) film. Note the onset and endpoints of the superconducting transition at approximately 5.2 and 5.1 K, respectively. Inset: Optical image of the substrate/film mounted on a chip package. The wire-bonded Al contacts are labeled A, B, C, D. Current was driven A-D, while the voltage drop was measured B-C.

**Supplementary Table 3. Offset characterization *via* 3.9 K measurements of superconducting V thin films.** Various measured 3.9-K resistances of Si/Si-N/V(100 nm) films. The results shown are averaged over 10-60 measurements, on two contact sets; the uncertainties listed are resulting standard deviations. The excitation current was 10 mA. Shown are *R*_ADBC_, *R*_DACB_ and their average (see text above for definitions), both without (left) and with (right) the preamplifying channel scanner.

|  | ***R*_ADBC,_**  **_w/o preamp_**  **(μΩ)** | ***R*_DACB,_**  **_w/o preamp_**  **(μΩ)** | ***R*_average,_**  **_w/o preamp_**  **(μΩ)** | ***R*_ADBC,_**  **_w/ preamp_**  **(μΩ)** | ***R*_DACB,_**  **_w/ preamp_**  **(μΩ)** | ***R*_average,_**  **_w/ preamp_**  **(μΩ)** |
| --- | --- | --- | --- | --- | --- | --- |
| Contact Set #1 | -0.4 ± 0.6 | -0.3 ± 0.6 | -0.4 ± 0.4 | 5.6 ± 0.2 | -4.1 ± 0.3 | 0.8 ± 0.2 |
| Contact Set #2 | 0.3 ± 0.5 | 0.7 ± 0.6 | 0.5 ± 0.4 | 6.1 ± 0.1 | -5.63 ± 0.05 | 0.24 ± 0.06 |


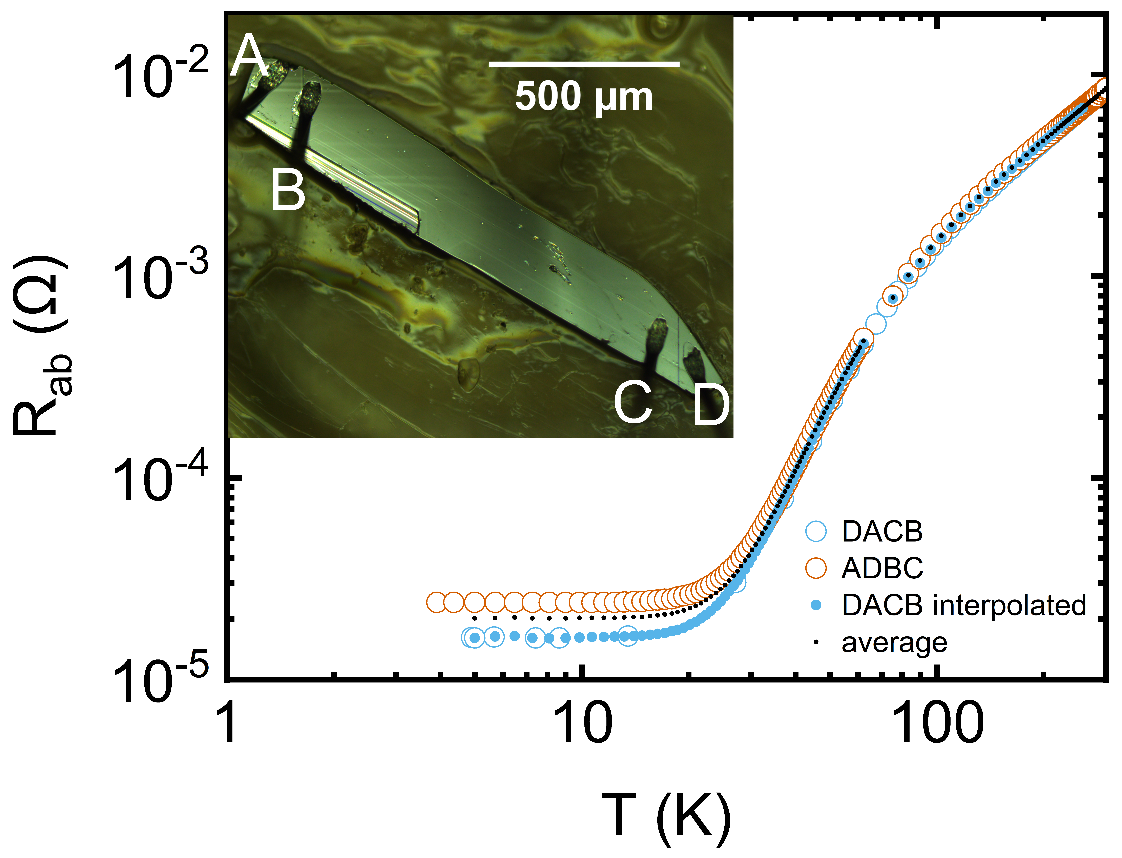


**Supplementary Fig. 5. Offset correction of PdCoO_2_ ρ_ab_(*T*) measurements.** *R*_ADBC_ and *R*_DACB_ *vs*. *T* for the CVT-grown crystal in Figs. 3a,b. (A, B, C, and D label the contacts, as shown in the image). The measurements were made with 10 mA excitation with the preamplifying channel scanner. After interpolation of *R*_DACB_(*T*) to generate data at identical temperatures to *R*_ADBC_(*T*), the two data sets were averaged (see the above text for detailed justification), resulting in the black data points, upon which Figs. 3a,b, are based.


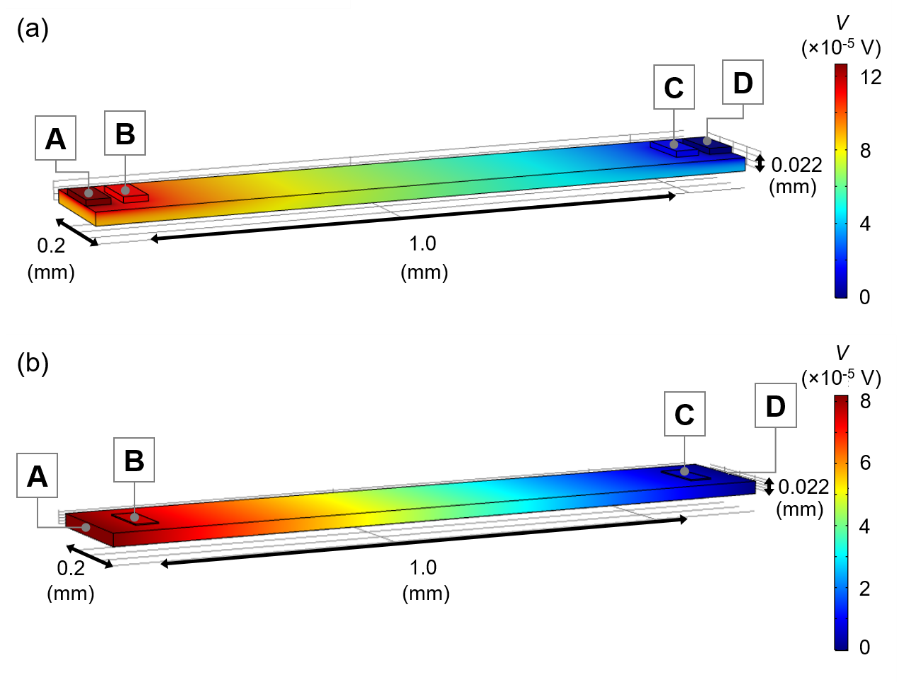


**Supplementary Fig. 6. Finite element simulations of current flow and voltage drop in PdCoO_2_ crystals.** Simulations were performed with COMSOL Multiphysics on crystals with dimensions (0.022 × 1.000 × 0.200 mm^3^), designed to closely resemble the crystal in Figs. 3a,b. Panel (a) is for a geometry with all top contacts, while (b) is for a geometry with side current contacts. The contacts are labeled A, B, C, D, an excitation current of 10 mA was used (injected at A and extracted at D), and the relevant resistivity parameters are discussed in the text and shown in Supplementary Table 4. Contacts B and C are the voltage contacts, placed 1 mm apart, edge-to-edge. The resulting simulated 300-K voltage drops are shown as color maps based on the scale to the right. Note the through-thickness color gradient near the contacts in (a).

**Supplementary Table 4. Input resistivity values and extracted resistances from finite element simulations.**

Shown are 300-K and residual low-*T* values of the PdCoO_2_ *a-b* plane resistivity, *c*-axis resistivity, their ratio, and the Cu contact resistivity^13,16,17^, which were used as inputs for the simulations. As described above, the PdCoO_2_ values shown are from metathesis/flux-grown crystals^13,16^, measured on focused-ion-beam patterned samples for the *a-b*-plane case^16^. The last two columns show the output from such simulations, specifically the simulated four-wire resistance (*R*_sim_) in the top-current-contact and side-current-contact geometries. Corresponding PdCoO_2_ RRR values are shown in the bottom row.

|  | **ρ_ab_**  **(Ω cm)** | **ρ_c_**  **(Ω cm)** | **ρ_c_/ρ_ab_** | **ρ_Cu_**  **(Ω cm)** | ***R*_sim, top contact_ (mΩ)** | ***R*_sim, side contact_**  **(mΩ)** |
| --- | --- | --- | --- | --- | --- | --- |
| 300 K | 3.05×10^-6^ | 1.03×10^-3^ | 338 | 1.67×10^-6^ | 10.2 | 7.03 |
| Low-*T* | 8.1×10^-9^ | 6.0×10^-6^ | 741 | 7×10^-12^ | 0.0367 | 0.0193 |
| RRR | 376 | 171 |  |  | 278 | 364 |

**Supplementary Table 5. Simulation parameters reproducing the observed RRR ratio in top-contact measurements.** Shown are analogous parameters to Supplementary Table 4, with the exception that the input low-*T* (residual) value of ρ_ab_ (bolded) has been selected to reproduce the measured top-contact RRR of ~440. The deduced residual ρ_ab_ of 4.44 nΩ cm generates a top-contact RRR of 436, which the simulations suggest requires a side-current-contact (true) RRR of 670 (rightmost column).

|  | ***ρ*_ab_**  **(Ω cm)** | ***ρ*_c_**  **(Ω cm)** | ***ρ*_c_/*ρ*_ab_** | ***ρ*_Cu_**  **(Ω cm)** | ***R*_sim, top contact_ (mΩ)** | ***R*_sim, side contact_ (mΩ)** |
| --- | --- | --- | --- | --- | --- | --- |
| 300 K | 3.05 × 10^-6^ | 1.03 × 10^-3^ | 338 | 1.67 × 10^-6^ | 10.2 | 7.03 |
| Low-*T* | **4.44 × 10^-9^** | 6.0 × 10^-6^ | 1350 | 7 × 10^-12^ | 0.0234 | 0.0105 |
| RRR | 687 | 171 |  |  | 436 | 670 |

**Supplementary Note 4. ICP-MS Trace Impurity Analysis Details**

As noted in the main text, 54 elements were selected for ICP-MS trace impurity analysis of both metathesis/flux- and CVT-grown PdCoO_2_ crystals. The selection of these 54 elements was based on their being either known to form ABO_2_ compounds, or being known impurities in the growth reagents. A full listing of these elements, and their corresponding analyte and analysis mode is provided in Supplementary Table 6. Elements that are known to form at least one ABO_2_ compound were first identified, in part using Supplementary Ref.^18^. Purity certifications for the commercial reagents used in the crystal growth processes were then examined. Supplementary Table 7 shows the grades and predominant impurities of the reagents used in most crystal growth batches in this work, including the crystals that were used for ICP-MS and transport measurements. The least pure reagent, Pd, is >99.995% pure, corresponding to <55 ppm impurities. Based on Supplementary Table 7, the detected presence of Ni, Mn, Fe, Sn, Ir, and Ag in the final PdCoO_2_ crystals (see Tables 2-4) is thus expected at some level. Additionally, Pt would not be surprising as an impurity in a Pt-group reagent element such as Pd, and the presence of Ni, Mn, Fe, and Zn in the reagents suggests that the first-row transition metals Cu and Cr would also not be unexpected. All the elements in Tables 2-4 can thus be reasonably accounted for based on the reagents, with the exception of Al. The latter may arise from Al-containing sample handling apparatus, particularly weighing boats.

**Supplementary Table 6. Tested elements and ICP-MS analytical conditions**

Analytical conditions for all the trace elements quantified in this work by ICP-MS. The analyte for each element is listed in the second column, with the corresponding analysis mode in the third column. Kinetic energy discrimination helium collision cell (KED-He) or triple quadrupole oxygen collision cell (TQ-O_2_) mode were used.

| **Element** | **Analyte** | **Analysis mode** |  | **Element** | **Analyte** | **Analysis mode** |
| --- | --- | --- | --- | --- | --- | --- |
| Li | ^7^Li | KED-He |  | Pd | ^105^Pd | TQ-O_2_ |
| B | ^11^B | KED-He |  | Ag | ^107^Ag | TQ-O_2_ |
| Na | ^23^Na | KED-He |  | In | ^115^In | KED-He |
| Mg | ^24^Mg | KED-He |  | Sn | ^118^Sn | KED-He |
| Al | ^27^Al | KED-He |  | Ba | ^138^Ba | KED-He |
| Si | ^29^Si | KED-He |  | La | ^139^La | TQ-O_2_ |
| K | ^39^K | KED-He |  | Ce | ^140^Ce.^16^O | TQ-O_2_ |
| Ca | ^44^Ca | KED-He |  | Pr | ^141^Pr.^16^O | TQ-O_2_ |
| Sc | ^45^Sc.^16^O | TQ-O_2_ |  | Nd | ^144^Nd.^16^O | TQ-O_2_ |
| Ti | ^49^Ti | KED-He |  | Sm | ^149^Sm.^16^O | TQ-O_2_ |
| V | ^51^V.^16^O | TQ-O_2_ |  | Eu | ^153^Eu | TQ-O_2_ |
| Cr | ^52^Cr.^16^O | TQ-O_2_ |  | Gd | ^157^Gd.^16^O | TQ-O_2_ |
| Mn | ^55^Mn | KED-He |  | Tb | ^159^Tb.^16^O | TQ-O_2_ |
| Fe | ^57^Fe | KED-He |  | Dy | ^163^Dy.^16^O | TQ-O_2_ |
| Co | ^59^Co | TQ-O_2_ |  | Ho | ^165^Ho.^16^O | TQ-O_2_ |
| Ni | ^60^Ni | KED-He |  | Er | ^166^Er.^16^O | TQ-O_2_ |
| Cu | ^63^Cu | KED-He |  | Tm | ^169^Tm.^16^O | TQ-O_2_ |
| Zn | ^66^Zn | KED-He |  | Yb | ^172^Yb | TQ-O_2_ |
| Ga | ^71^Ga | KED-He |  | Lu | ^175^Lu.^16^O | TQ-O_2_ |
| As | ^75^As.^16^O | TQ-O_2_ |  | W | ^182^W | KED-He |
| Rb | ^85^Rb | KED-He |  | Re | ^185^Re | KED-He |
| Sr | ^88^Sr.^16^O | TQ-O_2_ |  | Os | ^189^Os | KED-He |
| Y | ^89^Y.^16^O | TQ-O_2_ |  | Ir | ^193^Ir | TQ-O_2_ |
| Zr | ^90^Zr.^16^O | TQ-O_2_ |  | Pt | ^195^Pt | TQ-O_2_ |
| Mo | ^98^Mo.^16^O | TQ-O_2_ |  | Au | ^197^Au | TQ-O_2_ |
| Ru | ^101^Ru | TQ-O_2_ |  | Hg | ^202^Hg | TQ-O_2_ |
| Rh | ^103^Rh | TQ-O_2_ |  | Tl | ^205^Tl | KED-He |

**Supplementary Table 7. Reagent grades and impurities**

Purity grades and predominant impurity elements (> 1 ppm) from the manufacturers’ certificates of analyses for the commercial reagents used to grow the crystals characterized here by ICP-MS.

|  | **Manufacturer** | **Reagent grade** | **Predominant impurity elements found** |
| --- | --- | --- | --- |
| Co_3_O_4_ | Alfa Aesar | 99.9985% (≤ 15 μg/g) | Fe, Mn, Ni, Ca, Zr, Mg, Zn |
| PdCl_2_ | Thermo Fisher Scientific | 99.999% (≤ 10 μg/g) | Rh |
| Pd | Sigma-Aldrich | 99.995% (≤ 55 μg/g) | Ag, Fe, Ca, Sn, As, Ir, K, Mo |

**Supplementary References**

1. Shannon, R. D., Rogers, D. B. & Prewitt, C. T. Chemistry of noble metal oxides. I. Syntheses and properties of ABO_2_ delafossite compounds. *Inorg. Chem.* **10**, 713–718 (1971).

2. Harada, T. Thin-film growth and application prospects of metallic delafossites. *Mater. Today Adv.* **11**, 100146 (2021).

3. Harada, T., Ito, S. & Tsukazaki, A. Electric dipole effect in PdCoO_2_/β-Ga_2_O_3_ Schottky diodes for high-temperature operation. *Sci. Adv.* **5**, eaax5733 (2019).

4. Renner, H. *et al.* Platinum Group Metals and Compounds. in *Ullmann’s Encyclopedia of Industrial Chemistry* 1–73 (Wiley, 2018).

5. Oranskaya, M. A. & Mikhailova, N. A. Dissociation pressure and vapour pressure of palladium chloride. *Russ. J. Inorg. Chem.* **5**, 5–7 (1960).

6. Binnewies, M., Glaum, R., Schmidt, M. & Schmidt, P. *Chemical Vapor Transport Reactions*. (De Gruyter, 2012).

7. Chemical Rubber Company. *CRC Handbook of Chemistry and Physics*. (CRC Press, 2022).

8. Prewitt, C. T., Shannon, R. D. & Rogers, D. B. Chemistry of noble metal oxides. II. Crystal structures of platinum cobalt dioxide, palladium cobalt dioxide, copper iron dioxide, and silver iron dioxide. *Inorg. Chem.* **10**, 719–723 (1971).

9. Mackenzie, A. P. The properties of ultrapure delafossite metals. *Reports Prog. Phys.* **80**, 032501 (2017).

10. Villars, P. & Cenzual, K. PdCoO_2_ Crystal Structure: sd_1920856 data sets. in *PAULING FILE in: Inorganic Solid Phases*. https://materials.springer.com/isp/crystallographic/docs/sd_1920856 (2016).

11. Wexler, A. & Corak, W. S. Superconductivity of vanadium. *Phys. Rev.* **85**, 85–90 (1952).

12. Rogers, D. B., Shannon, R. D., Prewitt, C. T. & Gillson, J. L. Chemistry of noble metal oxides. III. Electrical transport properties and crystal chemistry of ABO_2_ compounds with the delafossite structure. *Inorg. Chem.* **10**, 723–727 (1971).

13. Takatsu, H. *et al.* Roles of high-frequency optical phonons in the physical properties of the conductive delafossite PdCoO_2_. *J. Phys. Soc. Japan* **76**, 104701 (2007).

14. Hicks, C. W. *et al.* Quantum oscillations and high carrier mobility in the delafossite PdCoO_2_. *Phys. Rev. Lett.* **109**, 116401 (2012).

15. Tanaka, M., Hasegawa, M. & Takei, H. Growth and anisotropic physical properties of PdCoO_2_ single crystals. *J. Phys. Soc. Japan* **65**, 3973–3977 (1996).

16. Nandi, N. *et al.* Unconventional magneto-transport in ultrapure PdCoO_2_ and PtCoO_2_. *npj Quantum Mater.* **3**, 66 (2018).

17. Kasap, S. O. *Principles of Electronic Materials and Devices*. (McGraw-Hill Education, 2018).

18. Sunko, V. Angle resolved photoemission spectroscopy of delafossite metals. (Max Planck Institute for Chemical Physics of Solids, 2019).
